# Effect of Active Ingredients of Chinese Herbal Medicine on the Rejuvenation of Healthy Aging: Focus on Stem Cells

**DOI:** 10.1155/2020/7307026

**Published:** 2020-07-08

**Authors:** Chen Wang, Shuang Ling, Jin-Wen Xu

**Affiliations:** Institute of Interdisciplinary Medical Science, Shanghai University of Traditional Chinese Medicine, Shanghai 201203, China

## Abstract

Stem cells (SCs) are special types of cells with the ability of self-renewal and multidirectional differentiation. As the organism ages, the ability to maintain homeostasis and regeneration deteriorates and the number and activity of stem cells decline. Theoretically, the restoration of stem cells might reverse aging. However, due to their own aging, donor-derived immune rejection, and difficulties in stem cell differentiation control, a series of problems need to be solved to realize the potential for clinical application of stem cells. Chinese herbal medicine is a nature drug library which is suitable for the long-term treatment of aging-related diseases. Modern pharmacological studies have revealed that many active ingredients of Chinese herbal medicines with the effect of promoting stem cells growth and differentiation mainly belong to “reinforcing herbs.” In recent years, exploration of natural active ingredients from Chinese herbal medicines for delaying aging, improving the stem cell microenvironment, and promoting the proliferation and differentiation of endogenous stem cells has attracted substantial attention. This article will focus on active ingredients from Chinese herbs-mediated differentiation of stem cells into particular cell type, like neural cells, endothelial cells, cardiomyocytes, and osteoblasts. We will also discuss the effects of these small molecules on Wnt, Sonic Hedgehog, Notch, eNOS-cGMP, and MAP kinase signal transduction pathways, as well as reveal the role of estrogen receptor *α* and PPAR *γ* on selectively promoting or inhibiting stem cells differentiation. This review will provide new insights into the health aging strategies of active ingredients in Chinese herbal medicine in regenerative medicine.

## 1. Introduction

Stem cells are undifferentiated cells capable of self-renewal to produce unlimited cells of the same type, as well as being able to differentiate into other cell types. During differentiation, stem cells gradually lose their pluripotency and become specialized cells with a more specialized function. Compared with embryonic stem cells, adult stem cells exist in highly differentiated tissues, which dedifferentiate and replace dead and damaged cells under appropriate conditions. Adult stem cells include neural stem cells (NSCs), hematopoietic stem cells (HSCs), bone marrow mesenchymal stem cells (BMSCs), epidermal stem cells (ESCs), and adipose-derived stem cells (ADSCs). The multipotential characteristics of stem cells may provide beneficial strategy for age-related diseases treatment.

With aging, the ability to maintain body homeostasis and regenerate damaged tissues decreases, resulting in the occurrence of age-related diseases. As humans age, metabolism, self-renewal, differentiation, or quiescent state of endogenous stem cells are damaged and become exhausted. The stem cell niche, as the in vivo microenvironment where stem cells reside, changes with age, which limited the tissue regeneration [1, 2]. Because of the attenuation of adult stem cells regenerative potential in the elderly, the reduced benefits of autologous stem cell therapy and the immune rejection of other donors have become obstacles to stem cell transplantation therapy [3]. If we can provide correct small molecules intervention and proper survival microenvironment for ameliorating the potential of aging stem cell regeneration in tissue repair, it will improve the efficiency of endogenous stem cell-mediated tissue healing mechanism.

Chinese herbal medicine has a long history of treating aging-related diseases. Modern medical research has revealed that many active ingredients of Chinese herbal medicines with the characteristics of “Tonifying-Qi,” “Tonifying-Kidney,” and “Tonifying-Blood” have the effect of promoting the growth and differentiation of stem cells. As a complementary approach, the active ingredients of traditional Chinese medicine target specific signal pathways and epigenetic processes, offering a powerful tool for manipulating cell fate to achieve the desired effect.

This envisages that Chinese herbal medicine treatment will become a rejuvenation strategy for healthy aging, which is beneficial to improve the microenvironment of stem cells in vivo. It also promotes the autonomous and intrinsic signaling pathways of proliferation and differentiation, as well as the repair of damaged tissue by endogenous stem cells.

## 2. Effect of Chinese Medical Herbs on Stem Cell Differentiation

The active ingredients of traditional Chinese medicine are mostly small molecular compounds, which are attractive approaches to control the stem cell fate. The biological effects of small molecules are fast and dose-dependent, allowing precise control of specific pathological situations. The small molecules are easier to handle and administrate, which makes them more practical for clinical applications and therapeutic development compared to genetic interventions. Chemical regulation of cell fate provides a wide range of applications in delaying stem cell aging and promoting tissue and organ regeneration. Small molecules of traditional Chinese medicine can target endogenous stem cells and enhance their self-renewal, expansion, differentiation, and viability in regenerative medicine. A summary list of stem cell differentiation induced by active small molecules of Chinese herbal medicine is shown in Table 1.

### 2.1. Neural Differentiation

**Figure 1:**
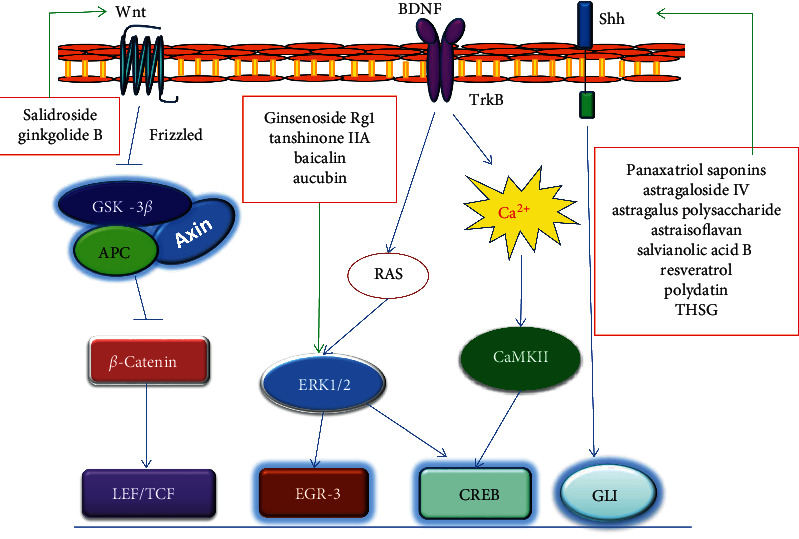
Schematic diagram of signaling in neural cell differentiation induced by active ingredients of traditional Chinese medicine. BDNF: brain-derived neurotrophic factor; Shh: sonic hedgehog.

Numerous studies have shown that the active ingredients of Chinese medical herbs have the effect of promoting nerve cells differentiation. Ginsenoside Rg1 treatment of mouse embryonic stem cells and human adipose-derived stem cells induced a significant increase in neuron-like cell populations in a time- and dose-dependent manner and upregulated the mRNA or protein expression of neuronal-specific neurofilament (NEFM), neural cell adhesion molecule (NCAM), synapsin-1 (SYN-1), and *β*-tubulin III, respectively [4, 5]. Saponins derived from Panax notoginseng also had neurodifferentiation promoting effects similar to ginsenosides [6, 7]. Salvianolic acid B and tanshinone IIA are active substances from the root of *Salvia miltiorrhiza* Bunge, which is widely used as a traditional Chinese medical herb for stroke. In vitro, salvianolic acid B improved the differentiation of neurospheres into neuronal lineage and further promoted the outgrowth of neurites and differentiated into neurons [8]. Tanshinone IIA treatment also induced neuronal differentiation in three cell models, immortalized C17.2 neural progenitor cells, rat embryonic cortical NSCs, and rat PC12 pheochromocytoma cells, in a dose-dependent manner [9, 10]. Baicalin is a flavonoid found in the Chinese herb *Scutellaria baicalensis*. Previous studies had demonstrated that baicalin could promote the neural differentiation of human iPS cells, immortalized mouse C17.2 neural progenitor cells, and rat embryonic cortical NSCs [11–13]. Interestingly, baicalin improved neural differentiation but inhibited glial formation [12]. Many other traditional Chinese medical herbal ingredients also have the ability to differentiate stem cells into specialized cell subtypes. Recent studies revealed that astragaloside IV, astragalus polysaccharide, and astraisoflavan, three effective active substances of *Astragalus propinquus* Schischkin, could induce the differentiation of NSCs into dopamine neurons and promote the mRNA expression of dopaminergic neuron-specific tyrosine hydroxylase and dopamine transporter in vitro [14]. Ginkgolide B is a biologically active terpenic lactone present in Ginkgo biloba. Li et al. [15] demonstrated that Ginkgolide B promoted cell cycle withdrawal and neuronal differentiation of adult NSCs in the subventricular zone (SVZ) after birth of the mouse lateral ventricle. Natural lignans and iridoid compounds including aucubin facilitated neuronal differentiation and neurite outgrowth in NSCs from the rat embryonic hippocampus or in rat neuronal hippocampal cell line HiB5 cells [16, 17]. Salidroside, a glucoside of tyrosol found in the plant *Rhodiola rosea* Linn., could induce rat BMSC differentiate into cholinergic nerve cells in vitro. When salidroside stimulated rat BMSCs, the expression levels of neuron-specific enolase (NSE), brain-derived neurotrophic factor (BDNF), *β*-tubulin III, and glial fibrillary acidic protein (GFAP) on the 6th day; the positive rates of NSE, microtubule-associated protein-2 (MAP-2), *β*-tubulin III, and GFAP in the immunofluorescence staining images on the 3th day; and the acetylcholine (Ach) positive rate on the 3rd, 6th, and 9th days were significantly higher than those of the control group [18]. The detailed signaling pathways of active ingredients inducing neural cell differentiation are shown in Figure 1.

### 2.2. Endothelial Cell and Cardiomyocyte Differentiation

**Figure 2:**
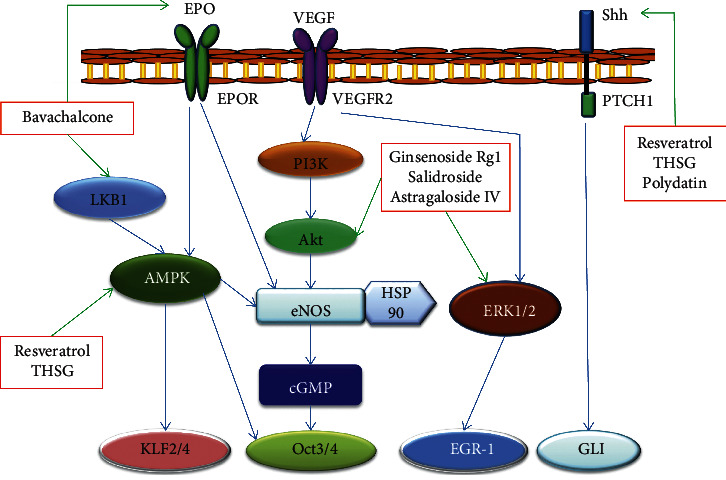
Schematic diagram of signaling in endothelial cell differentiation induced by active ingredients of traditional Chinese medicine. EPO: erythropoietin; VEGF: vascular endothelial growth factor; Shh: sonic hedgehog.

Circulating endothelial progenitor cells (EPCs) may contribute to vasculogenesis after ischemia and tissue injury, so studies have been conducted to investigate the function of EPCs in ischemic diseases. Our previous studies have found that bavachalcone, an isopentenyl chalcone from *Psoralea corylifolia* Linn., promoted the differentiation of EPCs and neovascularization in vivo [19]. In a series of cell-based experiments, Tang and his colleagues observed that ginkgolide B, icariin, tanshinone IIA, astragaloside IV, ginsenoside Rg1, and salidroside exert angiogenic endothelial differentiation effects of human bone marrow-derived EPCs [20–24]. A study reported that salvianolic acid A could augment EPC numbers and promote EPC migration, adhesion, and the vasculogenesis capacity in myocardial ischemia-reperfusion (I/R) rat model [25]. In another study, monotropein, an iridoid monoterpenoid, promoted mobilization and differentiation of bone marrow-derived EPCs and attenuated cell autophage and apoptosis, finally, improving wound healing [26]. Polysaccharide, also called glycan, presents protective effects on cell damage. *Astragalus* polysaccharide and *Morinda officinalis* oligosaccharide have been shown to stimulate the proliferation and differentiation of EPCs by releasing growth factors through the paracrine pathway [27, 28]. The detailed signaling pathways of active ingredients inducing endothelial cell differentiation are shown in Figure 2.

**Figure 3:**
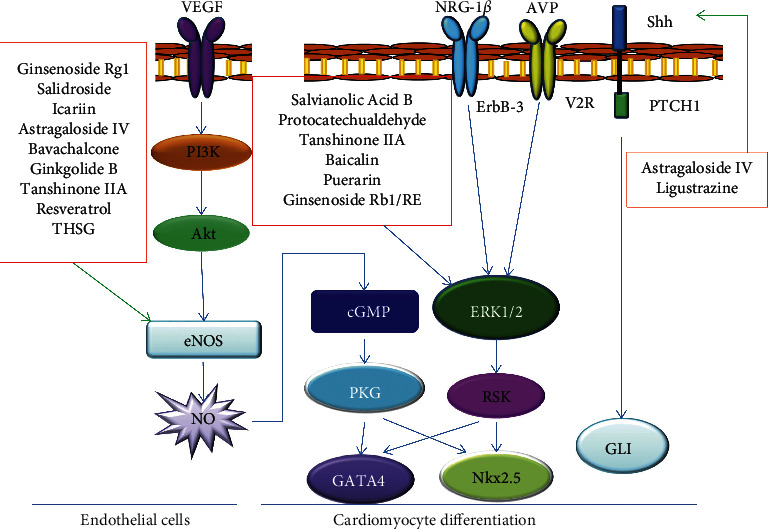
Schematic diagram of signaling in cardiomyocyte differentiation induced by active ingredients of traditional Chinese medicine. VEGF: vascular endothelial growth factor; NRG-1*β*: neuregulin-1*β*; AVP: arginine vasopressin; Shh: sonic hedgehog.

Cardiomyocytes are the chief cell type in the heart. Embryonic stem cells (ESCs) remain a potential source for cardiomyocyte replacement. Several studies from the Lou team indicated that icariin has a role in promoting the differentiation of mouse embryonic stem cells into cardiomyocytes with heartbeat function [29–31]. Ginsenoside Rb1 (panaxadiol) and ginsenoside Re (panaxatriol) treatment upregulated the expression of mesodermal and cardiac transcription factor genes in the early stage of differentiation induction and cardiac sarcomeric genes in the late stage of differentiation maturation [32]. The ginsenoside Rb1 and Re also elevated the expression of potassium voltage-gated channel subfamily E member 1 (KCNE1). Ginsenoside Re treatment showed a longer beating duration compared to the control [32]. Salvianolic acid B alone had little effect, but costimulation with vitamin C or transforming growth factor beta 1 (TGF-*β*1) in a concentration-dependent manner promoted differentiation of embryonic or BMSCs and increased expression of cardiomyocyte maturation markers [33, 34]. Similarly, salvianolic acid B, protocatechualdehyde, and tanshinone IIA induced human placenta-derived mesenchymal stem cells to differentiate into cardiomyocytes and increased the expression of GATA4, atrial natriuretic peptide (ANP), *α*-actin, and troponin I to differing degrees [35]. In addition, continuous baicalin treatment promoted the differentiation of embryonic stem cells and increased the proportion of *α*-actinin-positive cardiomyocytes and transcript level of cardiac specific markers, such as *α*-myosin heavy chain (*α*-MHC), ventricular myosin light chain-2 isoform (MLC-2v), and atrial natriuretic peptide (ANP) [36]. Furthermore, puerarin, another flavonoid compound, significantly increased the number of mouse embryonic stem cell-derived cardiomyocytes, induced embryonic stem cells to differentiate into ventricular-like cells elevated typical cardiac marker expression, and presented complete electrophysiological signals [37]. An in vivo study also showed that simultaneous administration of stem cell transplantation with 2,3,5,4'-tetrahydroxystilbene-2-O-*β*-d-glucoside (THSG) significantly reduced S-T segment elevation, increased heart rate compared with the myocardial infarction group, and upregulated expression of Nkx2.5, GATA-4, and connexin 43 in myocardial tissue [38]. The detailed signaling pathways of active ingredients inducing cardiomyocyte differentiation are shown in Figure 3.

### 2.3. Osteoblast Differentiation

**Figure 4:**
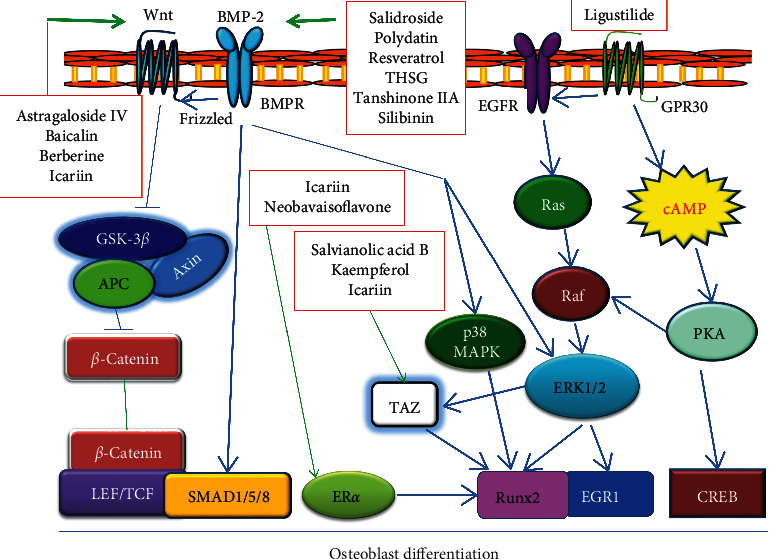
Schematic diagram of signaling in osteoblast differentiation induced by active ingredients of traditional Chinese medicine. BMP-2: bone morphogenetic protein-2.

Osteoblasts are bone forming cells. Stimulation of osteoblast differentiation from MSCs is an effective therapeutic strategy for bone repair. Several studies indicated that icariin, a flavonoid glycoside of *Epimedium brevicornu* Maxim., significantly promoted osteogenic differentiation by increasing ALP activity and Runx2, *β*-catenin, type I collagen (COLI), osteocalcin (OCN), and osteopontin (OPN) expression in rat BMSCs [39, 40]. Moreover, icariin had a bidirectional regulation effect on promoting the differentiation of bone marrow mesenchymal stem cells or bone marrow stromal cells into osteoblasts and inhibiting the differentiation into adipocytes [41, 42]. Micro-CT analysis showed that icariin relieved the loss of cancellous bone of the distal femur in OVX mice [41]. Kaempferol, the main active component of *Rhizoma Drynariae*, also showed the effect of stimulating osteogenic differentiation [43, 44]. Interestingly, according to Chinese medicine theories, *Epimedium brevicornu* Maxim and *Rhizoma Drynariae* both belong to kidney-tonifying herbs that act to strengthen bones. Ligustilide, an ingredient from *Angelica sinensis*, had the function in promoting osteoblast differentiation of preosteogenic cell line MC3T3-E1 and BMSCs and inducing the phosphorylation and activity of EGFR and ERK1/2, through the fast response pathway mediated by the estrogen membrane receptor GPR30 [45]. Accumulating results showed that a large number of Chinese herbal ingredients significantly promoted BMSC differentiation, such as salvianolic acid B at 5 *μ*M, tanshinone IIA at 1 and 5 *μ*M, polydatin at 30 *μ*M, salidroside at 0.5–10 *μ*M, puerarin at 10 *μ*M, ginkgolide B at *μ*M, THSG at 6.25–25 *μ*g/ml, catalpol at 50 *μ*M, and baicalin at 50 *μ*M [46–54], among which salvianolic acid B and polydatin can increase the ALP activity and upregulate the expression of osteogenic genes COLI, OPN, OCN, Runx2, osterix, and DLX5 in human BMSCs [46, 47]. The detailed signaling pathways of active ingredients inducing osteoblast differentiation are shown in Figure 4.

## 3. Signaling Pathways in Stem Cell Differentiation Activated by Chinese Medicinal Herbal Ingredients

Stem cell differentiation is usually controlled by cell signaling. Here, we discuss the signaling involved in stem cell differentiation activated by Chinese herbal small molecules. Targeted signaling pathways for active small molecules are also shown in Figures 1–4.

### 3.1. Wnt/*β*-Catenin Signaling

Wnt signaling pathway regulates stem cell differentiation and proliferation. *β*-Catenin transcriptional activity is dependent on Wnt signaling, which can be regulated by a variety of Chinese herbal ingredients. For example, icariin activated the Wnt/*β*-catenin signaling pathway during the differentiation of osteoblasts [41, 55], and the same pathway was provoked by ginkgolide B and salidroside during neuronal differentiation [15, 56]. In addition, in the process of promoting osteoblast differentiation, tanshinone IIA, polydatin, and catalpol also selectively activated the bone morphogenetic proteins (BMP)/Wnt signaling pathway [47, 48, 53]. Surprisingly, salvianolic acid B promoted bone marrow-derived mesenchymal stem cells to differentiate into alveolar endothelial cell type I and hepatocytes in the same Wnt pathway [57, 58].

### 3.2. Shh/Gli1 Signaling

Sonic hedgehog (Shh) signaling is involved in many types of stem cell differentiation. A large number of studies have revealed that whether stem cells develop into cardiomyocytes [59, 60] and vascular endothelial cells [61–63] or differentiate into neurons [64–66] and osteoblasts [67, 68], the Shh signaling pathway plays an important role. Astragaloside IV promoted mesenchymal stem cells into neuronal cells [69], endothelial cells [22], and cardiomyocytes [70]. The angiogenesis and cardiomyocyte survival induced by astragaloside IV in rats with acute myocardial infarction attributed to upregulation of the gene expression of Shh pathway and their activity of receptors and signal transducers [71]. Astragaloside IV also promoted the proliferation and migration of osteoblast-like cells through the Shh pathway [72]. Gao et al. reported [14] that astragaloside IV, astragalus polysaccharide, and astraisoflavan all promoted the proliferation and committed differentiation of neural stem cells into dopamine neurons. The mechanism of these active ingredients of radix astragali included the Shh signaling pathway. Panaxatriol saponins not only improved the neurological function and reduced infarct volume in middle cerebral artery occlusion (MCAO) rats but also enhanced cerebral perfusion, capillary density, and angiogenesis in ischemic border areas after MCAO surgery and upregulated VEGF and Ang-1 expression by activating the Shh signaling pathway [73]. A study of salvianolic acid injection revealed that salvianolic acid has the effect of improving stroke via Shh signaling [74]. The role of the Shh pathway involved in improving brain function, increasing neural progenitor cell (NPC) proliferation, and promoting the long-term survival of new neurons in the subventricular zone (SVZ) was determined by intraperitoneal injection of salvianolic acid injections for 14 days after 24 hours of stroke onset. Upregulation of nuclear translocations of Shh, Ptch, and Gli1 was observed in the area around the infarction, accompanied by the massive production of brain-derived neurotrophic factor (BDNF) and nerve growth factor (NGF) [74]. Multiple studies have reported that resveratrol protected nerve damage in ischemic stroke by activating the Shh/Gli1 signaling pathway [75]. Interestingly, polydatin, a resveratrol glycoside, also showed the same effect and mechanism [76]. Furthermore, the preparation of *Polygonum multiflorum*, mainly containing 2,3,5,4'-tetrahydroxystilbene-2-O-*β*-D-glucoside (THSG, another resveratrol glycoside), promoted hair growth by stimulation of Shh expression [77]. Chinese wolfberry is used as a dual-use fruit for herbal medicine and food. Its polysaccharide, *Lycium bararum* polysaccharides, has been reported to improve the differentiation of hippocampal NSCs [78]. *Lycium bararum* polysaccharides also play a role in reducing apoptosis and oxidative stress by regulating glycogen synthase kinase-3*β* (GSK-3*β*) phosphorylation, Shh, and phosphoinositide 3-kinase (PI3K)/Akt signaling pathways [79]. A report indicated that atractylenolide III has the effect on inducing differentiation of mesenchymal stem cells into chondrocytes, enhancing the expression of cartilage-associated proteoglycans, transcription factor Sox9, and chondrogenic markers, as well as significantly increasing expression of Shh signal and its target gene Gli1 [80]. To sum up with western medical concepts, these active ingredients, which come from “Invigorating-Qi” herbs and “Invigorating-blood” herbs according to traditional Chinese medicine theory, can achieve the effect of regulating immunity and bone marrow function through the induction of Shh signal pathway.

### 3.3. Notch/Jagged Signaling

In addition to activating Wnt signaling, salvianolic acid B also inhibited Notch receptor Notch1/3, Notch ligand Jagged2, and Notch receptor target Hes1/5 expression in promoting the differentiation of human embryonic stem cells into hepatocytes [57]. Another study showed that serum containing matrine inhibited the proliferation of rat hepatocyte progenitor cell WB-F344 and the expression of Jagg1 and HES1 protein in a concentration- and time-dependent manner, indicating that matrine-induced differentiation of WB-F344 cells through the Notch cell signaling pathway [81]. Salidroside inhibited the proliferation of D1 cells, induced the phenotype of neurons, and upregulated the expression of neuron-specific markers, such as eno2/NSE, microtubule-associated protein-2, and tubb3/*β*-tubulin III, which were related to downregulation of the expression of Notch1 and its downstream target protein Hes1 [82]. Astragaloside IV is known to have neuroprotective property. A study found that in vitro, astragaloside IV induced neural stem cells to differentiate into neuronal marker *β*-tubulin III (+) cells and astrocyte marker GFAP (+) cells. Astragaloside IV treatment resulted in an increase in the number of *β*-tubulin III (+) cells in the hippocampus of rat Alzheimer's disease models transplanted with neural stem cells and improvements in learning and memory [83]. In addition, osthole, a natural coumarin derivative from *Cnidium monnieri* (L.) Cuss, also increased the number of neurons in hippocampal DG and CA3 regions, significantly improved the learning and memory function of mice with mechanical brain injury, and upregulated the expression of self-renewal genes Notch1 and Hes1 [84]. As a phytoestrogen, icariin increased the expression and activity of estrogen receptor 1 (ER*α*), and this effect of icariin on the differentiation of BMSCs into osteoblasts was blocked by the estrogen nuclear receptor antagonist ICI 182780 [39, 41, 55, 85]. Icariin facilitated osteogenesis in ovariectomized rats by inhibiting peroxisome proliferator-activated receptor *γ* (PPAR*γ*), CCAAT/enhancer-binding protein *α* (C/EBP*α*), and fatty acid binding proteins 4 (FABP4) mRNA expression, and downregulating Jagged1 protein expression in bone tissue [86]. In addition, transcriptional coactivator TAZ modulated both osteoblast and adipocyte differentiation from mesenchymal stem cells by stimulating the activities of RUNX2 [87] and suppressing the activities of peroxisome proliferator-activated receptor-gamma (PPAR*γ*) [88]. Studies demonstrated that icariin stimulated the activation of TAZ as evidenced by increased total TAZ protein and nuclear translocation in the osteogenic differentiation [40, 89]. Similar to icariin, kaempferol fortified the activity of TAZ by enhancing RUNX2-mediated osteoblast differentiation and suppressing PPAR*γ*-stimulated adipocyte differentiation [43]. More studies have confirmed that two coumarins isopsoralen and psoralen, four flavonoids isobavachalcone, bavachin, corylifol A, and neobavaisoflavone, and one meroterpene phenol bakuchiol of *Psoralea corylifolia* [90], glycinol of *Glycyrrhiza uralensis* [91], notoginsenoside R1 of *Panax notoginseng (Burk.)* [92], and puerarin of *Puerariae Lobatae Radix* [93] had the activity of phytoestrogens, which activated estrogen receptor signaling and promoted the differentiation of bone marrow mesenchymal stem cells or mouse embryonic osteogenic precursor cells into osteoblasts.

The eNOS/NO/cGMP pathway plays an important role in the differentiation of osteoblasts, cardiomyocytes, and EPCs. In induced osteogenic differentiation, icariin stimulated Akt phosphorylation, enhanced nitric oxide synthase 3 (eNOS) and protein kinase G (PKG) expression, increased nitric oxide (NO) production, and elevated soluble guanylyl cyclase (sGC) and cyclic guanosine monophosphate (cGMP) levels [94]. Icariin also promoted the expression of genes involved in cardiac development and enhanced the increase in endogenous NO production in stem cells [95]. Curcumin, in the same manner of icariin, significantly promoted the differentiation process of embryonic stem cells and increased the gene expression and protein levels of cardiac specific transcription factors NKX2.5, cardiac troponin I, myosin heavy chain, and eNOS. Incubation of cells with curcumin resulted in a dose-dependent increase in intracellular nitrite and elevated levels of intracellular cGMP [96]. Additionally, salvianolic acid B also showed similar effects in inducing nitric oxide production during osteogenic differentiation [97]. Treatment with ginkgolide B and salidroside resulted in cell proliferation, angiogenesis, and differentiation of BMSCs-EPCs; enhanced the ability of EPCs to integrate into vascular networks; and activated Akt phosphorylation and NO production [21, 25]. Studies have reported that osteogenic differentiation of BMSCs is closely related to the activation of the mitogen-activated protein (MAP) kinase signaling pathway and the upregulation of transcription factors Runx2 and Dlx5. Various Chinese herbal ingredients rely on the MAP kinase signaling system in regulating stem cell differentiation, such as icariin [39, 98], salidroside [99], salvianolic acid B [46], and tanshinone IIA [100]. The activity of p38 MAP kinase can control stem cell differentiation switch between neurogenesis and cardiomyogenesis [101], which is one of the mechanisms of icariin in inducing cardiomyocyte differentiation [102].

Furthermore, AMP-activated protein kinase (AMPK) signaling pathway is also involved in bavachalcone-induced differentiation of EPCs and ginsenoside RH 2 (s)-stimulated differentiation of osteoblasts [19, 103]. Resveratrol enhanced the expression of pluripotency of mouse embryonic stem cells and increased the pluripotency markers Oct3/4, Sox2, Nanog, and Klf4 by activating the AMPK/Ulk1 pathway [104]. Likewise, THSG, a glycoside of resveratrol, enhanced self-renewal of human dental pulp stem cells via an AMPK signaling pathway [105]. Evidences showed that medicarpin and cryptotanshinone promoted the differentiation of C3H10T1/2 mesenchymal stem cells into brown adipocytes by increasing the expression of thermogenesis marker uncoupling protein 1 (UCP1), upregulating brown fat-specific markers, and reducing the expression of white fat markers, which were associated with the activation of AMPK pathway [106, 107].

## 4. Roles of Wnt, Shh, and Notch Signaling Pathways in Stem Cell Senescence and Effects of Active Ingredients from Traditional Chinese Medicine

The antiaging effects of ER*α*, eNOS/NO/cGMP, and AMPK pathways have been reported in a large number of high-quality reviews, so it will not be detailed here. In this article, we compare the effect of active ingredients of traditional Chinese medicine on Wnt, Shh, and Notch signaling pathways in stem cell differentiation and cell senescence. Wnt, Shh, and Notch signaling not only regulate stem cell differentiation but also participate in cell senescence. Multiple reports suggested that persistent chronic stimulation and dysfunction of Wnt signaling increased cell senescence, mitochondrial biogenesis, and reactive oxygen species (ROS) production [108–111]. Recent study reported that LiCl activated the Wnt/*β*-catenin pathway and promoted the senescence of mouse neural stem cells; ginsenoside Rg1 inhibited the activation of the Wnt/*β*-catenin pathway and promoted the proliferation of neural stem cells and hematopoietic stem/progenitor cells [112, 113]. In another aging mouse model induced by d-galactose, ginsenoside Rg1 prevented oxidative stress, and glutathione peroxidase (GSH-px) and malondialdehyde (MDA) inhibited phospho-histone H2A.X, 8-OHdG, p16 (Ink4a), Rb, p21 (Cip1/Waf1), and p53 in senescent Sca-1⁺ hematopoietic stem/progenitor cells [113]. However, Shh acting as an endogenous antiaging factor suppresses endometrial stem cell aging [114]. Shh gene delivery also inhibited radiation-induced cell senescence in the salivary glands of mice [115].

Some researchers found that compared with young mice (5 weeks old), the expression of Shh decreased in osteoblasts but increased in osteoclasts in old mice (60 weeks old), which is closely related to senile fracture healing [116], due to colocalization of Shh and Gli1 with osteogenic markers Runx2 and Osx, both of which can be observed during fracture healing [117]. Accumulated evidence indicated that resveratrol and two of its glycosides, polydatin and THSG, could activate the Shh pathway [75–77] and delay cell senescence [118–122]. During endothelial cell senescence, Notch expression was enhanced and activated [123, 124], and Notch signaling further mediated secondary senescence and inflammation in oncogene-induced senescence [125, 126]. Salidroside was one of the active ingredients of traditional Chinese medicine that not only blocked Notch signaling [82] but also inhibited the replicative cell senescence [127, 128].

## 5. Conclusion

Throughout the effect of active ingredients from Chinese herbal medicine on stem cells, the following points can be summarized: (1) the botanical source of these active ingredients is Chinese herbal medicines with the characteristics of “Tonifying-Qi,” “Tonifying-Kidney,” and “Tonifying-Blood,” which are often used to treat aging-related diseases; (2) the same active ingredient induces stem cells to differentiate into different tissue cells; although the active ingredients are different, the signal pathways through which they act are similar; (3) the same active ingredient may require different auxiliary conditions in the differentiation of different tissue cells; for example, salvianolic acid B stimulates differentiation into cardiomyocytes that requires vitamin C or TGF*β*, which is not necessary to differentiate into osteoblasts; (4) several studies have confirmed that there are crosstalk and integration among Wnt, Shh, Notch, and other signaling pathways in regulating stem cell differentiation; for example, salvianolic acid B activated Wnt signaling but prevented Notch signaling when promoting the differentiation of embryonic stem cells into hepatocytes [57]; salvianolic acid B also activated the Shh signaling pathway and promoted functional recovery and neurogenesis in neuroprotection [74]; during the promotion of osteoblast differentiation, the function of salvianolic acid B was also involved in nitric oxide-cGMP pathway [97]; (5) Wnt, Shh, and Notch are not only signal pathways of stem cell differentiation but also important factors of cell senescence. As shown in Table 1 and Figures 1–4, the targeted cells and activated pathways of active ingredients of Chinese herbal medicine are summarized. Nevertheless, more research is necessary to explain the targeted molecules of these active ingredients of Chinese herbal medicine.

## Figures and Tables

**Table 1:** Small molecule compounds from Chinese medical herbs inducing stem cell differentiation.

Active ingredients	Differentiated cells	Stem cells or model	Pathways	Effects	References
Astragaloside IV	Endothelial cell-like cells	Rat mesenchymal stem cells	N/A	1. Differentiated into endothelial cell-like cells and promoted tube formation in vitro 2. Upregulated the expression of Cx37, Cx40, and Cx43 and enhanced gap junctional intercellular communication (GJIC) function	[22]
	Neuronal cells	Rat neural stem cells	Sonic hedgehog	1. Differentiation into dopamine neurons 2. Promoted the expressions of Shh, Nurr1, and Ptx3 mRNAs	[14]

Astraisoflavan	Neuronal cells	Rat neural stem cells	Sonic hedgehog	1. Differentiation into dopamine neurons 2. Promoted the expressions of Shh, Nurr1, and Ptx3 mRNAs	[14]

Aucubin	Neuronal cells	Rat neural stem cells and neural precursor cells	N/A	1. Promoted lengthening and thickness of axons and remyelination at 3 weeks after sciatic nerve injury 2. Promoted differentiation of neural precursor cells into GABAergic neurons	[16, 17, 129]

Baicalin	Cardiomyocytes	Murine embryonic stem cells	N/A	1. Increased the proportion of *a*-actinin-positive cardiomyocytes 2. Upregulated cardiac specific genes *a*-MHC, MLC-2v, and ANP	[36]
	Neuronal cells	Neural stem cells derived from the cortex of embryonic E15-16 SD rats	N/A	1. Increased the percentages of mature neuronal marker MAP-2-positive staining cells and decreased glial marker GFAP staining cells 2. Downregulated the expression of *p*-stat3 and Hes1 but upregulated the expressions of NeuroD1 and Mash1	[13]
	Osteoblasts	N/A	Wnt/*β*-catenin signaling	1. Increased significantly the osteoblastic mineralization levels of mRNAs encoding the bone differentiation markers OCN, OPN, and COL-1	[54]

Bavachalcone	Endothelial cells	Rat bone marrow mesenchymal stem cells and rat hindlimb ischemia model	ROR*α*-erythropoietin-AMPK axis	1. Promoted rat bone marrow-derived cells to differentiate into EPC significantly 2. Stimulated blood flow recovery in ischemic hindlimbs, increased circulating EPC, and promoted capillary neovascularization	[19]

Berberine	Osteoblasts	Bone marrow-derived mesenchymal stem cells	Wnt/*β*-catenin signaling	Promoted osteogenic differentiation and osteogenic genes Runx2, OPN, and OCN expression	[132]

Catalpol	Osteoblasts	Bone marrow mesenchymal stem cells	Wnt/*β*-catenin signaling	1. Enhances the osteogenic differentiation 2. Significantly enhanced osteoblast-specific gene expression, alkaline phosphatase activity, and calcium deposition	[53]

Curcumin	Cardiomyocytes	Human embryonic stem cells	NO-cGMP signaling	1. Promoted differentiation into cardiomyocytes 2. Significantly increased the gene expression and protein levels of NKX2.5, cTNI, MHCs, and eNOS	[96]

Ginkgolide B	Endothelial progenitor cells	Human bone marrow	Akt/eNOS and p38 MAPK signaling	1. Promoted proliferation and endothelial gene expression, significantly enhanced VEGF-induced migration response, and improved the vascular network composition of EPCs 2. Induced phosphorylation of eNOS, Akt, and p38	[20]
	Neuronal cells	Neural stem cells derived from mouse subventricular zone (SVZ)	Wnt/*β*-catenin	1. Promoted neuronal differentiation 2. Increased the level of nuclear *β*-catenin and activated the Wnt pathway	[15]
	Osteoblasts	Rat bone mesenchymal stem cells and MC3T3-E1 cells	Wnt signaling	1. Promotes osteoblast differentiation 2. Reduced OVX-induced bone loss by enhancing osteoblast activity	[51]

Ginsenoside Rb1/RE	Cardiomyocytes	Human embryonic stem cell	N/A	1. Enhanced differentiation into cardiomyocytes 2. Upregulated Nkx2.5, Tbx5, MHC, and KCNE1 expression	[32]

Ginsenoside Rg1	Neuronal cells	Mouse embryonic stem cells and human adipose-derived stem cells	Wnt/*β*-catenin pathway, MEK-ERK1/2, and PI3K-Akt signaling	1. Promoted cell proliferation and neural phenotype differentiation 2. Upregulated the mRNA or protein expression of NSE, MAP-2, NEFM, NCAM, synapsin-1, and *β*-tubulin III	[4, 5]

Ginsenoside RH2 (S)	Osteoblasts	MC3T3-E1 cells	PKD/AMPK signaling	1. Stimulated osteoblastic differentiation and mineralization 2. Enhanced the expression of Runx2, ALP, OCN, OPN, Osx, and ColI	[103]

icariin	Cardiomyocytes	Mouse embryonic stem cells	p38 MAPK pathway in early differentiation and NO-cGMP signaling	1. Facilitated the directional differentiation of ES cells into cardiomyocytes 2. Elevated PGC-1-alpha, PPAR-alpha, and NRF-1 expression in early differentiation 3. Increased mRNA level of MHC, MLC-2v, *α*-actinin, and troponin T	[29–31, 95, 102]
	Osteoblasts	Rat bone mesenchymal stem cells	ER*α*-Wnt/*β*-catenin signaling, RhoA-TAZ signaling, and AKT-eNOS-cGMP pathway	1. Significantly enhanced osteogenic differentiation and increased ALP activity and Lef1, TCF7 DLX5, OPN, OCN, COLI, ER*α*, CXCR4, and HIF-1*α* expression 2. Upregulated TAZ, Runx2, *β*-catenin, OPN, and Dlx5 expression mainly at the early stage and OCN expression at the late stage 3. Improved osteoporosis, inhibited the expression of PPAR*γ*, C/EBP*α*, FABP4 mRNA, N1ICD, and Jagged1 proteins and increased Notch2 mRNA in OVX rats	[39–42, 55, 86, 89, 94]

Kaempferol	Osteoblasts	Rat bone mesenchymal stem cells	Interaction between TAZ and RUNX2	Promoted physical interaction between TAZ and RUNX2 to increase osteoblast differentiation of mesenchymal cells	[43]

Ligustilide	Osteoblasts	MC3T3-E1 cells and rat bone mesenchymal stem cells	GPR30/EGFR pathway	1. Promoted osteoblast differentiation 2. Activated phosphorylated EGFR and ERK1/2	[45]

Myricetin	Osteoblasts	Human bone marrow stem cells and human periodontal ligament stem cells	Wnt/*β*-catenin pathway, BMP-2/Smad, and ERK/JNK/p38 MAPK	1. Enhanced osteogenic differentiation 2. Upregulated BMP-2 3. Increased mRNA expression of OCN, COL-1, ALP, and RUNX2	[130, 131]

Polydatin	Osteoblasts	Human bone marrow stromal cells and OVX mouse model	BMP-2-Wnt/*β*-catenin signaling	1. Significantly improved the proliferation and calcium deposition of hBMSCs and markedly stimulated the expression of the mRNAs RUNX2, OPN, DLX5, *β*-catenin, TAZ, and OCN 2. Maintained the bone matrix in the OVX mouse model	[47]

Puerarin	Osteoblasts	Rat bone marrow stromal cells	Estrogen receptor-dependent manner	1. Enhanced osteoblast differentiation 2. Increased ALP activity, OCN, and Wnt5b	[50, 93]
Quercetin	Osteoblasts	Human adipose tissue-derived stromal cells, mouse adipose stem cells, rat mesenchymal stem cells, and rat bone marrow-derived mesenchymal stem cells	p38 MAPK, ERK1/2 and JNK MAPK signaling	1. Promoted the osteogenic differentiation 2. Promoted expressions of ALP, Osx, Runx2, BMP-2, TGF-*β*1, Col-1, OPN, and OCN	[134–137]

Resveratrol	Neuronal-like cells	Human bone marrow mesenchymal stem cells and human cord blood-derived mesenchymal stem cells	Sonic hedgehog signaling, PKA-GSK3*β*, and *β*-catenin signaling	1. Differentiated into neuronal-like cell types 2. Significantly increased expression of the neuronal-specific marker genes Nestin, Musashi, CD133, GFAP, NF-M, MAP-2, and KCNH1 3. Increased expressions of Smo and Gli1 proteins	[75, 138–140]
	Osteoblasts	Mouse embryonic stem cells, rat adipose-derived mesenchymal stem cells	AMPK/Ulk1 pathway, and Sirt-1/Runx2 deacetylation	1. Enhancing osteogenic differentiation and mineralization 2. Enhanced expression of pluripotency markers Oct3/4, Sox2, Nanog, Klf4, SSEA-1, and ALP 3. Increased expression of Runx2 and decreased expression of PPAR-*γ*	[104, 141, 142]

Salidroside	Neuronal cells	Rat bone marrow mesenchymal stem cells, mouse mesenchymal stem cells	Notch and BMP signaling pathways	1. Inhibited the proliferation, increased expression level of NSE, BDNF, MAP2, *β*-tubulin III, GFAP, Wnt3a, *β*-catenin, LRP6, and Axin 2. The positive rate of Ach was significantly higher on the 3rd, 6th, and 9th day than on the 1st day	[18, 56, 82]

Salvianolic acid a/B	NF-M (+) neurons and NG2 (+) oligodendrocyte precursors	Neural stem cells derived from the cerebral cortex of embryonic mice, bone marrow-derived neural stem cells, and induced pluripotent stem cells	PI3K/AKT/GSK3*β*/*β*-catenin pathway	1. Promoted the neurite outgrowth of neural stem cells and their differentiation into neurons 2. Induced BDNF production	[8, 143, 144]
	Osteoblasts	Human mesenchymal stem cells and rat bone marrow stromal stem cells	ERK signaling and NO-cGMP signaling	1. Significantly promoted mineralization 2. Increased ALP activity, Runx2, osterix, OPG, and OCN level and the OPG/RANKL ratio	[46, 97]
	Hepatocytes	Human embryonic stem cells	Through upregulation of WNT pathway and inhibition of Notch pathway	1. Promoted hepatocyte differentiation and increased expression of albumin, tyrosine aminotransferase (TAT), CYP3A4, CYP2C19, UGT1A6, UGT1A8, and UGT2B7 2. Enhanced expression of TCF3 and LEF1 and downregulated Jagged2, and Hes1/5	[57]
	Alveolar epithelial cells type I	Rat bone marrow mesenchymal stem cells	WNT pathway	1. By day 14, the majority of bone marrow mesenchymal stem cells were morphologically differentiated into alveolar epithelial cells 2. Significantly increased the T1*α* and AQP-5 protein levels	[58]

Silibinin	Osteoblasts	Human bone marrow stem cells	Activating BMP and RUNX2 pathways	1. Promoted ALP activity and mineralization in hBMSCs 2. Increased the mRNA expressions of COLI, ALP, OCN, osterix, BMP-2, and RUNX2	[133]

Tanshinone IIA	Neuronal-like cells	Rat bone marrow mesenchymal stem cells	N/A	Significantly upregulated the expression levels of Nestin, NeuN, and NF200 in the transplanted cells in the BMSCs + tanshinone IIA treatment rats compared among the groups	[10]
	Osteoblasts	Mouse bone marrow mesenchymal stem cells and human periodontal ligament stem cells	ERK1/2-dependent Runx2 induction and BMP-Wnt signaling	1. Enhanced ALP activity on day 7 and calcium content on day 24 in the process of TSA-induced osteogenesis of mouse bone marrow mesenchymal stem cells 2. Promoted both osteogenic differentiation and maturation of periodontal ligament stem cells	[48, 70, 100]

2,3,5,4'- Tetrahydroxy- stilbene-2-O-*β*-D-glucoside (THSG)	Osteoblasts	Rat mesenchymal stem cells	N/A	Promoted osteogenic differentiation and increased ALP activity and OCN expression	[52]

## Abbreviations

8-OHdG: 8-Hydroxy-2'-deoxyguanosine
*α*-MHC: *α*-Myosin heavy chain
Ach: Acetylcholine
ADSCs: Adipose-derived stem cells
Akt: Protein kinase B
ALP: Alkaline phosphatase
AMPK: AMP-activated protein kinase
ANP: Atrial natriuretic peptide
BDNF: Brain-derived neurotrophic factor
BMP: Bone morphogenetic proteins
BMSCs: Bone marrow mesenchymal stem cells
BrdU: Bromodeoxyuridine
cGMP: Cyclic guanosine monophosphate
EGFR: Epidermal growth factor receptor
eNOS: Nitric oxide synthase 3
EPCs: Endothelial progenitor cells
ER*α*: Estrogen receptor 1
ERK1/2: Extracellular signal-regulated kinase 1/2
ESCs: Embryonic stem cells
FABP4: Fatty acid binding proteins 4
FGF: Fibroblast growth factor
GFAP: Glial fibrillary acidic protein
GPR30: A G protein-coupled receptor for estrogen
GSH-px: Glutathione peroxidase
GSK-3*β*: Glycogen synthase kinase-3*β*
HSCs: Hematopoietic stem cells
KCNE1: Potassium voltage-gated channel subfamily E member 1
MAP-2: Microtubule-associated protein-2
MAP kinase: Mitogen-activated protein kinase
MCAO: Middle cerebral artery occlusion
MDA: Malondialdehyde
MLC-2v: Ventricular myosin light chain-2 isoform
NCAM: Neural cell adhesion molecule
NEFM: Neuronal-specific neurofilament
NGF: Nerve growth factor
NO: Nitric oxide
NPCs: Neural progenitor cells
NSCs: Neural stem cells
NSE: Neuron-specific enolase
OCN: Osteocalcin
OPN: Osteopontin
OVX: Ovariectomized
PI3K: Phosphoinositide 3-kinase
PKG: Protein kinase G
PPAR*γ*: Peroxisome proliferator-activated receptor-gamma
sGC: Soluble guanylyl cyclase
Shh: Sonic hedgehog
SYN-1: Synapsin-1
SVZ: Subventricular zone
TAZ: Transcriptional coactivator with PDZ-binding motif
TGF-*β*1: Transforming growth factor beta 1
THSG: 2,3,5,4'-Tetrahydroxystilbene-2-O-*β*-d-glucoside.
